# Jottings

**Published:** 1909-12-04

**Authors:** 


### JOTTINGS.

It has been decided to appoint a fourth resident medical officer at Swansea Hospital.

The Asylums Officers' Superannuation Bill has now been finally passed, the Commons having agreed to the amendments of the Lords.

Her Excellency the Countess of Aberdeen opened the annual sale of patients' work at the Royal Hospital for Incurables, Dublin, on December 2.

The Lord Mayor, President of the Hospital, accompanied by the Lady Mayoress, will open the Metropolitan Hospital after the recent alterations on Monday, December 20, at 3 p.m.

The Medway Guardians have adopted plans for a balcony at their infirmary, which will provide 35 beds, at a cost of £10 per bed, for the special treatment of cases of consumption.

The income of the Hospital Saturday Fund for this pear amounts to 19,734*l*., an increase of 50*l*. for the corresponding period of last year. The accounts for 1909 will be closed on January 10, 1910.

The Joint Isolation Hospital Board recently constituted in the Isle of Wight has decided to erect, at a cost of some £8,000, a large permanent isolation hospital for the treatment of infectious diseases. Its site will be near Newport (I.W.).

At the annual meeting of the subscribers to the Stirling Royal Infirmary on November 19, reference was made to the proposed extension scheme. The estimated cost of the new buildings is £12,600, for which £5,440 has already been promised.

The British Home and Hospital for Incurables, Crown Lane, Streatham, held a sale of the patients' work on Wednesday, November 24, at the hospital. The proceeds of the sale were given to the patients, who thus provided themselves with a little pocket-money.

A meeting was held in St. Austell Public Rooms on November 17 to consider the need for erecting a cottage hospital for St. Austell and district, Cornwall. A legacy of £500, less legacy duty, has been received with the condition that a hospital is established before July 1911.

Remarkable allegations against the officials of the Parkhurst Infirmary were made recently at a meeting of Guardians. It was alleged that it had been necessary to appoint a new superintendent nurse, because things had been allowed to drift and everyone had been able to do as he or she pleased.

At a meeting of the Bridgend Guardians, Dr. Wyndham Randall, the Board's medical officer, reported that it was absolutely necessary to establish a small emergency hospital at Maesteg, with its 30,000 industrial population. The Clerk was requested to report as to the powers of the Guardians in the matter of providing hospital accommodation.

An interesting sketch of his daily hospital life has been written by a patient (Mr. Frank Thomson) of the Hastings Infirmary, and is published in the local paper. It is always instructive to know what our patients think of us and our institutions, and this genial column of information should prove of real value to all readers who are interested in institutional affairs.

The work of the British Fire Prevention Committee for the session that has just commenced includes the preparation and issue of reports in respect to a number of doors and roller shutters that have been recently tested by the committee, and on fire extinguishers, including a petrol extinguisher. Regarding tests to be undertaken in the near future these include a further concluding series of door tests mainly relating to composite doors, and the matter of roofing materials will also be shortly having the attention of the committee.

